# Impact of a CBPR-informed physical activity intervention before and during COVID-19 among women from a disadvantaged neighborhood in Sweden

**DOI:** 10.3389/fpubh.2022.997329

**Published:** 2022-11-21

**Authors:** Rathi Ramji, Elisabeth Carlson, Anders Kottorp, Margareta Rämgård

**Affiliations:** Department of Care Science, Faculty of Health and Society, Malmö University, Malmö, Sweden

**Keywords:** community-based participatory research, community resilience, pandemic (COVID-19), social support, community empowerment

## Abstract

**Background:**

Public health practitioners have been striving to reduce the social gradient and promote physical activity among citizens living in disadvantaged neighborhoods. The emergence of the COVID-19 pandemic, which has affected these citizens extensively, has posed a significant challenge to efforts to maintain a physically active lifestyle. Thus, the aim of this study was to explore the impact of a CBPR-informed physical activity intervention before and during the COVID-19 pandemic from the perspective of women from a socially disadvantaged neighborhood.

**Methods:**

A total of 34 women participated in a CBPR-informed physical activity intervention previously developed in collaboration with lay health promoters and other citizens from the same neighborhood. Focus group discussions were conducted at four time points, namely, at baseline prior to the intervention, post-intervention, 6 months after the intervention ended, and during the COVID-19 pandemic. The data were analyzed using qualitative content analysis following an inductive approach.

**Results:**

In total, four themes emerged from the discussions: “Wavering between frustration and action,” “Shifting from prioritizing family needs to taking control of self,” “Between isolation and social support,” and “Restricted access to health-related knowledge vs. utilizing internalized knowledge”.

**Conclusion:**

The results of this study reveal that building on CBPR-informed health promotion initiatives has the potential to foster individual empowerment and assist during acute situations like the COVID-19 pandemic through mobilizing communities and their resources, which leads to increased community resilience and health. This study is regarded as unique in that it involves evaluation of a CBPR intervention that was initiated ahead of the pandemic and followed even during the pandemic.

## Introduction

The world has been confronting novel challenges such as the COVID-19 pandemic and an unexpected increase in non-communicable diseases (NCDs) (1). A fair share of the risk of NCDs seems to occur due to poor lifestyle including a decrease in physical activity (PA) and increase in unhealthy dietary practices (2). Despite the vital role PA plays in health promotion and disease prevention, physical inactivity has reached epidemic proportions globally (3). Health equity is relevant for PA since both physical inactivity and sedentary behaviors are influenced by social determinants; specifically, socially disadvantaged neighborhoods have lower access to PA than their counterparts (4). In addition, the COVID-19 pandemic has created a double burden on health, especially among citizens living in disadvantaged neighborhoods, as physical inactivity and mental illness have been exacerbated (3, 5). Previous research suggests that citizens in these neighborhoods require special support that is tailored to their needs to help them deal with the complexities of newly emerging diseases. Such support should also promote integration to society where healthcare providers can better understand and respond to the needs of marginalized citizens (5, 6).

Furthermore, research also shows that inequalities in health cannot merely be explained by differences in the individual characteristics of citizens living in a neighborhood since the social and contextual features of the neighborhood are also identified to play a role (5). Thus, the current situation demands reorientation of traditional public health practices and shifting the goals of health promotion from solely achieving individual lifestyle changes to a more broadened approach that includes addressing the social and environmental factors (7). The Ottawa Charter of Health Promotion suggests that health is created in the context in which individuals thrive and engage in everyday activities (8). Previous research also suggests that the context is not merely a location where an individual exists, rather an environment in which human social interactions are embedded (9). Thus, a sustainable form of health promotion can be achieved by facilitating health at a community level, where communities become empowered to use and shape their environment to solve problems relating to health. Such an approach is also regarded as a dynamic method to address disease prevention by integrating risk factors and improving quality of life (10). Community health promotion aims to address social, cultural, and environmental processes related to health by enhancing community participation and thus empowering communities within a defined geographic area to increase control over their health and life (11). In recent years, several health promotion initiatives have prioritized efforts to increase physical activity at a community level (12, 13). Enhancing community participation in health promotion makes it a collaborative process, creating an ideological shift. Such a research based on a partnership between community members and academicians has now become both essential and ethical (11).

In contrast to the traditional model in which an academic researcher drives all aspects of research on health promotion conducted in a community setting, a translational research approach known as community–academic partnership (CAP) exists. This paradigm integrates science and practice to improve health equity (14–16). Within the umbrella of CAP lies community-based participatory research (CBPR), an approach where citizens from communities take part in the research process with an equal involvement of both academic and community stakeholders throughout the research process starting from conceptualizing a research problem to final dissemination (17). CBPR is inspired by participatory action research (PAR), as coined by a German-American social psychologist Kurt Lewin (18), and also from participatory research science, as conceived by a Brazilian educator Paulo Freire (19).

The goal of this approach is to achieve community empowerment by actively involving community members in the research processes and assuring that the true needs of the community are effectively addressed (20). Empowerment is a central goal in the theory and practice of health promotion, not least in CBPR programs. Empowerment is the process of taking control over one's own situation focusing on multiple aspects including personal, social, economic, and political forces. CBPR is a participatory approach with a long-term commitment to social action, which is based on the liberatory educator Paulo Freire's approach that states that the cyclic process of knowledge transformation through reflection and action promotes critical consciousness and critical thinking, which, in turn, can foster democratic participation, leading to sustainable social transformation (21). According to Freire, critical consciousness means the ability to gain understanding of the key problems in their immediate environment, which facilitates the ability to change through acting on the problems illuminated by the understanding (21). Wallerstein et al. (22) defined CBPR as a collaborative effort by the community together with academic and other stakeholders, who gather and use research and data, built upon community strengths and priorities to adopt multilevel strategies to improve health and promote social equity. In contrast to top-down approaches, where much of the health intervention is predetermined, this approach has been fruitful in co-developing and implementing interventions in partnership with community members. Building trust between community members, academic researchers, and other stakeholders is key to achieve sustainable and equitable partnerships. Trust depends on the function of relationships between the members of the community, academic researchers, and other stakeholders. It is also depends on how community members connected in social networks. In contexts where growing inequalities drive ill health, a CBPR approach is built on trust and equal partnership with the community.

This approach is regarded as a means to broaden the horizons of traditional public health practices with new visions for improving community health and wellbeing (17). Previous research on disease and natural disaster management and health also showed that an approach driven together with the citizens could help mitigate stress, as well as protect the health and wellbeing of communities by promoting resilience and recovery (23, 24). The value of a well-established CBPR partnership between the citizens, stakeholders, and academic researchers, with its potential to strengthen civil society and citizens, particularly during acute situations like the COVID-19 pandemic, has been established in a few studies. These studies have showed that activities involving a CBPR approach strengthened the individual and collective resilience of participants while mitigating the adverse effects of the pandemic. It also seemed to be an appropriate means to enhance emergency preparedness and communicate risk to vulnerable populations (25–27).

Several CBPR physical activity interventions have been developed and evaluated around the world. However, CBPR interventions implemented in urban residential areas are sparse. Some of the existing CBPR interventions targeted specific groups such as elderly (28), cancer patients (29), members of a church congregation (30, 31), or students (32). These interventions were often quantitatively evaluated from the researchers' perspective and seldom explored experiences of participants over time (33).

A few community health promotion programmes conducted in Sweden do exist but are not common (34–36). Northern European states do have a well-established welfare sector, but given that the sector has been gradually shrinking, there is a growing gap between the citizens and government institutions providing services including social services and healthcare. Therefore, there is an urgent need to find new ways to close this gap as, for instance, the Swedish system cannot only rely on civil society to fill this gap. The administration in the Swedish state is decentralized in that the regional healthcare and local municipal authorities have the power to make local decisions and thus have the capacity to reduce social inequalities. Despite that, there are only few fieldworkers left in these organizations due to budget issues. To fill this gap, a community-based collaboration with local partners and NGOs is important. Academics have a prime role in facilitating such initiatives, advocating for disadvantaged communities. Since Sweden does not have strong communities, new models of working together in an equal partnership is essential. By integrating such an approach into the local governance system while also including citizens from the community in the decision-making process, efforts can be sustainable and also can be relied on even during crisis situations such as the pandemic. Such work will also add important knowledge to the international research community on how the CBPR approach can be applied in a welfare state with a relatively large public sector involvement compared with states with a larger private sector involvement such as the United States.

Based on this background, a CBPR approach was applied within a community health promotion programme, Equal Health. This programme was established in a socially disadvantaged neighborhood in southern Sweden in the year 2017 initiated by researchers from Malmö University together with the citizens from the neighborhood and other stakeholders from public, private, and non-profit organization sectors (37). This neighborhood in Malmö city in southern Sweden was among the areas regarded as highly vulnerable by the Swedish National Police Authority owing to issues such as low education levels, unemployment, high rate of criminality, and poor health among the inhabitants (38). Furthermore, the members in the neighborhood also live in social isolation and lack social context where they can interact regularly with others.

This programme was also established in accordance with the recommendation of a city-level initiative Malmö Commission inspired by the WHO report *Closing the Gap* (39). The main aim of the programme was to promote equal health in socially disadvantaged neighborhoods using an approach where both structure and content were defined by the communities living in a disadvantaged neighborhood (37).

The first step in the programme was the trust-building process, where researchers participated in local activities that happened in the neighborhood meeting places. The research team interacted and familiarized themselves with the community, in particular the local women network. Conversation held with communities living in the neighborhood by one of the authors showed that the citizens had mistrust in healthcare and social services and perceived themselves to be stigmatized when in contact with these organizations. Health-related information and support they received were not suitable owing to language and sociocultural barriers. The process of migration and socioeconomic situation led to physical and psychosocial health problems including lack of sleep, pain, stress, and poor physical health. Despite having mounting health needs, the citizens expressed that they did not have access to health-promoting activities, and those available in their near neighborhood were not affordable. This made it evident that these citizens were not adequately represented in the society and that their voices had seldom been heard.

In the second step in the CBPR health promotion programme, the academic researchers together with the fieldworkers from the municipality invited citizens from the neighborhood to the meeting places to participate in a future workshop (40). The future workshop is a method that emerged during the post-war period in Germany where a group of people gather to discuss social problems and develop solutions through collective decision-making. The residents in the neighborhood were sent an open invitation to attend the future workshop through notices posted in public areas and the municipality meeting places, as well as were reached out through different community groups, such as the local women network. The future workshop was conducted in 2016, where the citizens from the neighborhood discussed their needs with the academic researchers, the fieldworkers from the municipality, and collectively identified strategies to promote health. About 150 participants participated in the future workshop. The academic researchers facilitated the workshop together with and an Arabic-speaking interpreter. This local context, with a well-established collaboration between actors and the pre-existing network with the citizens in the neighborhood who frequented the established meeting places, was a basis to mobilize participants and plan for the future workshops. Through the future workshops, five problem areas emerged from the discussions with the citizens: (a) physical inactivity, (b) poor mental health, (c) lack of access to self-care, (d) poor oral health, and (e) lack of health literacy (41). Some of the citizens from the neighborhood also volunteered to become health promoters to help coordinate the activities within the programme. These representatives called lay health promoters (LHPs) were employed within the programme and were responsible for facilitating participant recruitment, language interpretation, and above all were instrumental in building trust between the research team and the citizens (37).

In the third step, the LHPs together with the research team, community members, and other stakeholders from the municipality, social care, primary care, pharmacy, property owners, and NGOs such as Red Cross and Save the Children created a CBPR model inspired by a model earlier developed by Wallerstein et al. (42) for planning collaboration and implementation of health-promoting initiatives focusing on the problem areas described earlier.

**Figure 1:**
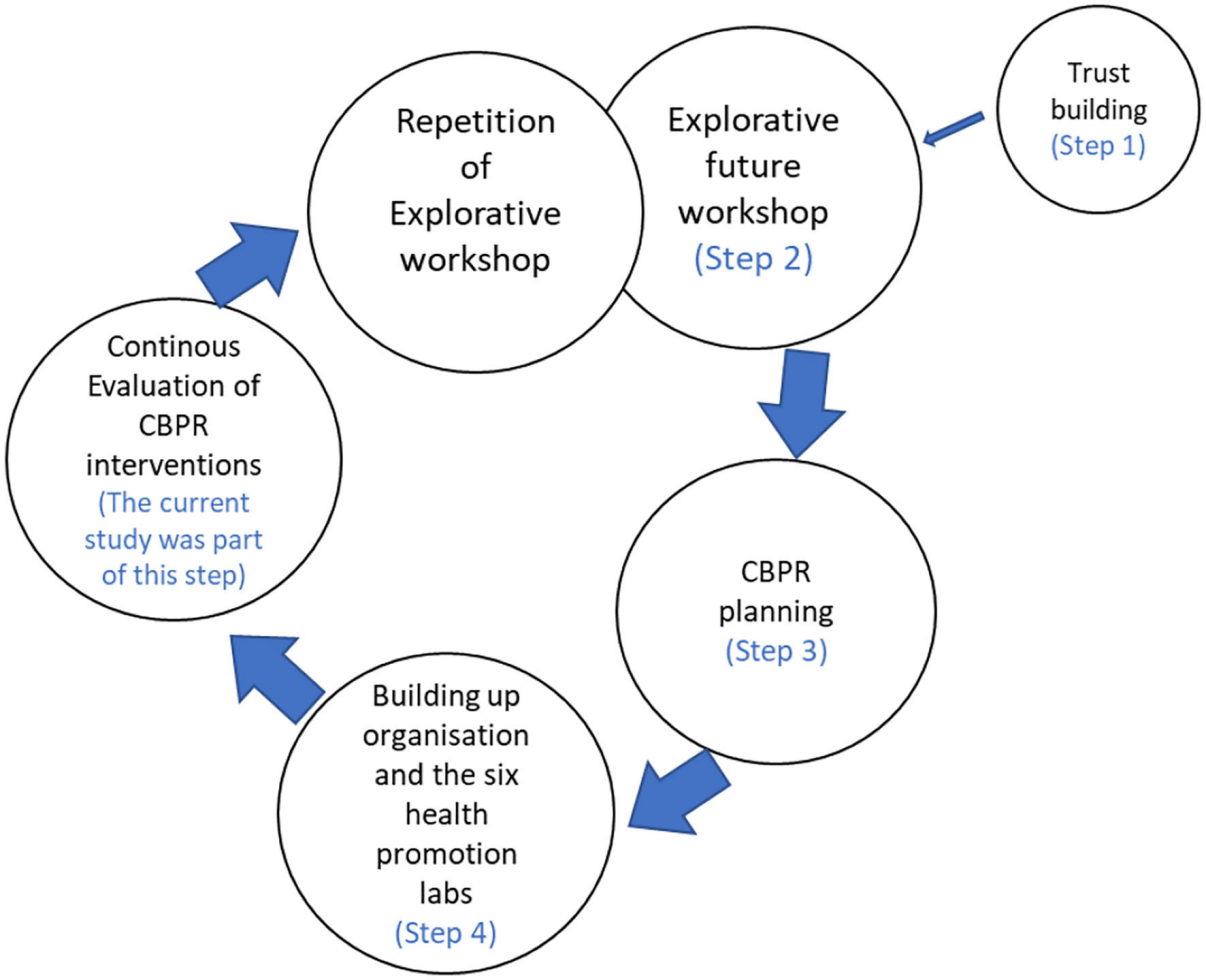
Steps within the Equal Health programme.

CBPR planning resulted in the development of six health-promoting co-creative labs focusing on problem areas raised in the future workshops such as oral health and diet, physical activity, mental health, women's health, social health, and safety in the area. These labs were driven by the citizens themselves and were facilitated by the LHPs. However, the LHPs worked across boundaries with various stakeholders to plan and manage the activities. The LHPs were also supported by a group of actors including the research team, with whom they shared and reflected on their experiences, and together developed strategies to address challenges. The LHPs were educated in CBPR methods and Freire's ideologies and were trained to manage power mechanisms, both at an individual level concerning their role in facilitating the activities and bringing together members of the community, and at the structural level with stakeholders (43). The different steps within the programme are presented in Figure 1.

**Figure 2:**
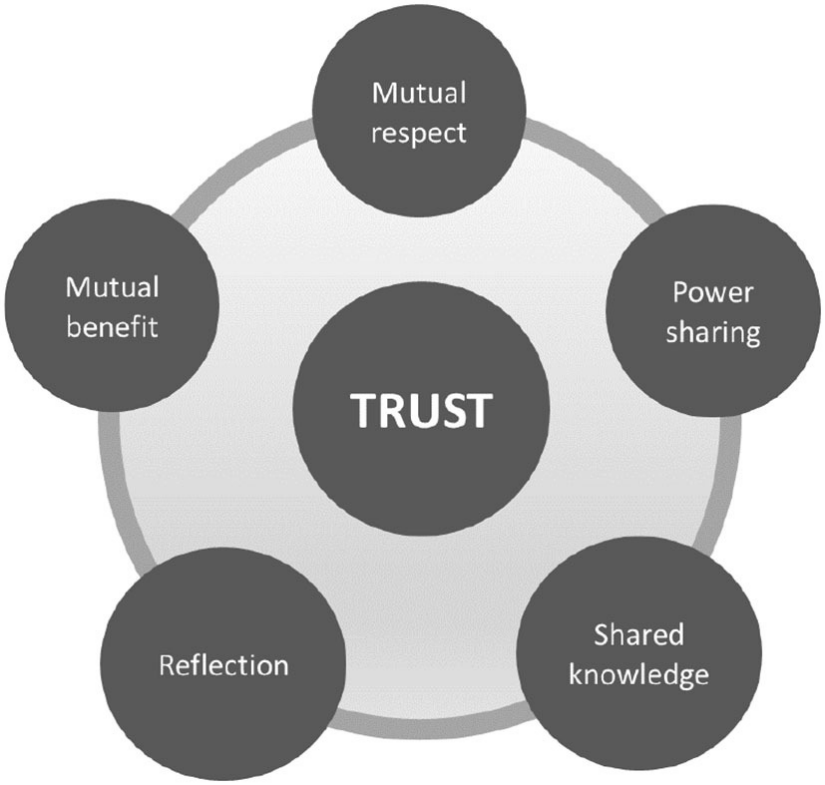
Values of the Equal Health programme.

The stakeholders or partners, LHPs, and community members who participated in CBPR planning met once every second week to plan, monitor, evaluate, and communicate the programme. The group also collectively defined a process for coordinating the activities and also periodically evaluating and developing them further in line with the citizens' needs. Furthermore, they worked around the values of the programme including mutual respect, mutual benefit, reflection, power-sharing, and knowledge mobilization (Figure 2). Mutual trust was considered central to all of these values. All the members had an opportunity to steer the proceedings by taking turns to be the meeting chairman. Dialogues at the meeting were the basis for various decisions. In case of disagreements, a voting process was initiated to ensure democratic action (41, 43). The partners and community members including the LHPs decided in the meetings to evaluate the health promotion programme in relation to the aforementioned values once every 6 months. This also included the evaluation of the activities in the individual co-creative labs.

All the activities within the programme were also followed by a strategical group, which comprised the vice chancellor of the university, director of the regional healthcare, representatives at the strategical levels from all stakeholders within the programme, and LHPs. The strategical group did not influence the proceedings of the programmes. They mobilized the knowledge from the programme and took it forwards to their organizations to work further with sustainable policy changes. Since the LHPs were also part of the strategical group, knowledge was transferred from this group back to the community.

A PA intervention programme, based on the needs and taking into account the varied capabilities of the community members, was established in the co-creative labs. The initial evaluation of the PA intervention in the co-creative lab showed the lack of activities exclusively for women in their near proximity, lack of affordable transportation to avail facilities elsewhere in the city, and lack of places to gather for group activities in their neighborhood (44). Thus, the PA intervention being evaluated in this study was offered cost-free and exclusively for women in the neighborhood. The evaluation of the intervention was an ongoing and iterative process. The physical activity intervention primarily intended to achieve reduced sedentary behavior and increase physical activity in everyday life among women in the neighborhood. In addition, the intervention did not target a particular aspect of health, but rather focused on the holistic view on health from the perspective of the participants. With the emergence of the pandemic, exploration of the experiences of participants before and during COVID-19 was warranted to understand the impact of the CBPR-informed PA intervention. Furthermore, experience from a prior epidemic has shown that when a new disease emerges and an acute situation arises, an already existing environment built on mutual trust can help improve understanding of disease control and suggest change that is reflective and community-sensitive without compromising on individual safety (45).

For nearly two decades, CBPR-based community health promotion has been proven to be an effective intervention approach in reducing inequalities (22, 46–49). Several CBPR physical interventions exist (28, 29, 50), some of which have also been initiated during the pandemic (51, 52). But only few interventions that initiated ahead of the pandemic followed through the pandemic, given that most parts of the world were under lockdown. However, since this study was based in Sweden, where no strict lockdown was imposed, there was an opportunity to evaluate the intervention even during the pandemic, which may give insights into the value-building CBPR work ahead of the emergence of a crisis situation.

*The aim of this study was to explore the impact of a CBPR-informed physical activity intervention before and during COVID-19 from the perspective of women from a socially disadvantaged neighborhood*.

## Methods

The current study reports a qualitative evaluation of a CBPR-informed PA intervention with a COVID-19 pandemic perspective. This was an exploratory study with an interpretative design. The participants were engaged in focus group discussions at four time points, ahead of the intervention (baseline), precisely after the intervention ended (post-intervention), 6 months after the intervention ended (long-term follow-up), and during the COVID-19 pandemic (during pandemic), which was about a year after the long-term follow-up.

### Context

The participants were citizens residing in one of the socially disadvantaged neighborhoods located in Malmö, one of the largest cities in southern Sweden (44). Nearly 40% of the population in this neighborhood comprises first- and second-generation migrants who are predominantly from Middle Eastern countries including Iraq and Syria, together with their families from other Arabic-speaking countries, such as Algeria, Egypt, Iran, Lebanon, Palestine, and Sudan (53).

### Participants

A total of 34 women aged 23–77 years were invited to participate in this study. All the participants in this study were non-Swedish-speaking migrants of Arabic descent. In this research, three LHPs were involved in contacting the women and facilitating the intervention and the focus groups. Given the results of the feasibility study (44), the intervention was exclusively performed in women as they were often isolated and physically inactive and as they did not have access to health-promoting activities that they could take part in the absence of men. For the focus group discussions held during the pandemic, the health promoters and the research team meticulously followed the recommendations of the Swedish Public Health Agency. The participants were requested to inform the LHPs if they experienced any flu symptoms on the day of the focus group discussion and were offered the possibility to reschedule the session. Of the 34 women in the group, about six of them could not participate in the focus group discussions held during COVID-19.

In this study, the participants were consistently informed *via* the LHPs that this study, which was part of the Equal Health programme, was built on the principles of openness, inclusion, and integrity. The trust-building process started with the strong partnership established between the research team and health promoters. The members of the research team participated in the physical activity intervention and were trained together with the group. Partnership between the participants was built based on trust and transparency. All the participants were informed that the decisions were to be collectively made and that all their thoughts were equally important. There was an ongoing process of reflection, and discussions regarding any conflicts that may emerge during the process were resolved at the end of every meeting. Although the participants were not familiar with other group members in the beginning, they were comfortable sharing their views. This was because they trusted the local health promoters who invited them to participate, who even facilitated the partnership between the members. Despite coming from different countries, the participants still shared the same language, followed similar sociocultural practices, perceived similar problems, and had similar goals, which facilitated and strengthened the group dynamics. This was in line with Etienne Wenger's view that a community of practice, where bringing together individuals sharing a similar concern to interact regularly, empowers the group and facilitates identifying collective solutions. This type of practice enables a collective responsibility where both reflections on their problems and solutions, and the action-taking process happen at the same time. This type of equitable collaboration enables connections that are beyond hierarchies and geographic boundaries (54).

### Community-based participatory research-informed intervention

This CBPR-informed PA intervention was developed by citizens from the neighborhood together with LHPs, one of whom was a physical activity enthusiast who was born and raised in the neighborhood. Following the CBPR planning and the establishment of the co-creative labs, 70 community members participated in a new workshop facilitated by the research team, where they together with the LHPs defined their expectations from a physical activity intervention. They also informed about personal, sociocultural, and structural factors that influenced the participation of community members in physical activity programmes. The discussions and reflections were condensed into specific action points. The citizens were particular about that the PA intervention should be tailored to individual capabilities. The citizens wanted the exercises to be related to their everyday activities and did not want it to involve the use of complex equipment that they could not afford. They also wanted to learn the right way to handle training tools and carry their body when performing everyday activities. They also wanted to gain knowledge on healthy diets and healthy mind. The citizens wanted the activities to be free of cost and happen in their near proximity.

A unique physical activity intervention was co-developed by the communities with the aim of building community and diversity together for a fair and equal fitness culture among citizens in the neighborhood based on their own perceived needs. The intervention had four key aspects focusing on natural human movements, nutrition and health, restoration and healing, and reflection session on why physical activity is important and should be available for all without differences. The fitness exercises focused on gradually facilitating a change in the participants' lifestyle, starting from simple body movements, which in due course evolved into more complex exercises customized to individual abilities. The participants in this programme also engaged in reflective dialogues regarding nutrition and the importance of eating fruits and vegetables. The intervention programme involved 10 sessions over a 3-month period, with one training session a week. The programme was coordinated by the LHPs. The programme was not merely a short-term intervention, but it also provided women the skills to become future health ambassadors who could spread the knowledge gained to others in their family and neighborhood.

Based on the results from the feasibility study (44), the intervention was offered two times a week over 3 months, so more participants had the opportunity to participate based on their convenience. In each session, which lasted for about 2 h, 15–20 participants were accommodated. All sessions concluded with a self-reflection. Evaluation of the intervention was an ongoing process where the participants were also actively engaged. The goal of the intervention was to evaluate the experiences of participants focused on broader aspects of health from their own perspective.

### Focus group interviews

The current study included data from 16 focus group discussions conducted over four time points. On the first three sessions, namely, baseline, post-intervention, and long-term follow-up, six to eight women per group engaged in focus group discussions. The last focus group discussion during the COVID-19 pandemic happened when recommendations against gathering in public spaces were temporarily lifted in early autumn of 2020. The focus group discussions happened in a large spacious room, where the participants were seated at a distance of 2 meter from each other. All the participants were requested to wash their hands ahead of the session. The participants were informed on all sessions that the discussions would be audiotaped and that the material would be used for research purposes only. Each focus group lasted 1–2 h, and discussions proceeded until no new information was identified. The interviews were primarily held in Swedish, while the health promoters translated back and forth between the participants who mainly spoke Arabic and the research team. The research team included an observer who was the second or third author or another PhD student from the programme (41) together with the first author. During the focus group discussions, the participants discussed between each other and together with the research team.

A CBPR interview guide previously developed by Wallerstein and colleagues (55) was used to initiate the discussion focusing on potential benefits of having participated in the community-based participatory research-informed PA intervention and understand the effect of collaboration with the health promoters and other group members during the activities. The CBPR interview guide focused on the context, group dynamics, equitable partnerships, intervention, and both health-related and structural outcomes of being part of the CBPR process including engaging in the intervention. These were also the domains explored in the focus group interviews. The questions on perceptions related to how their context and surroundings affected the participation in the group intervention and how they perceived the collaboration with the other participants, LHPs, and academic researchers were also asked to understand aspects that may hinder participant development. Further questions also explored the perceived outcomes related to the intervention. Additional questions related to the pandemic were also included in the last focus group discussion: (a) How have your lifestyle changed since the start of COVID-19 in the spring of 2020? (b) How has it been with following the healthy routines that you learned from your participation in health-promoting activities during the pandemic? (c) What kind of information about lifestyle changes related to the COVID-19 pandemic have you received?

### Analysis

The audiotaped data from the focus group interviews were transcribed verbatim, and the transcripts were analyzed using the inductive content analysis method guided by the approach of Elo and Kyngäs (56). As a first step, all transcribed interviews were meticulously read to identify text relevant to the aim of the study. Texts that were related to each other in terms of their content and context were grouped together. These interrelated texts known as meaning units were placed in a table for analysis. Later, the different meaning units were condensed into manageable texts. Finally, codes with names as close as possible to the original data were assigned to the condensed meaning units. Codes with similar content were grouped together into sub-themes. The sub-themes and codes were rechecked and compared with each other, as well as the original data. The overall main theme was identified at this stage, which summarized the information from all sub-themes earlier identified. The initial analysis was performed by the first author and the last author of this article and later verified by the second and the third author to increase the credibility of the study (57).

The results from this study were presented to the participants after the analysis was completed. This was carried out in a separate workshop in the presence of all the participants. The different themes were presented to the participants and reconfirmed if the research team had interpreted their thoughts in a meaningful way.

### Ethical considerations

The health promoters verbally informed all the participants in Arabic about the purpose of the research study prior to baseline focus group discussions as well as reminded them in the following two sessions. The participants were also assured that participation was voluntary and that they could leave the study at any point in time without any consequences.

The participants were contacted by the LHPs through a video call *via* WhatsApp and informed about the details of the study ahead of the focus group discussions during COVID-19. The participants were assured that all activities were carried out in accordance with guidelines from the Swedish Public Health Agency. The research group also ensured that there were no more than eight participants per group during the focus group interviews. The participants who preferred to avoid social contact due to COVID-19 and not participate in group discussions were offered the opportunity to be interviewed individually or through video conferencing. However, none of the participants desired this alternative.

All the aforementioned information was also provided to the participants in writing together with contact information of the research team both at baseline and when data were collected during COVID-19. The participants were asked to sign an informed consent form at baseline, as well as when they were invited to the focus group discussions during COVID-19. All data collected were anonymized and kept confidential. The data were only accessible to the members of the research team. The Swedish Ethical Review Authority approved this study (DNR 2018-382 and DNR 2020-04063).

## Findings

The participants' experiences in the participation in the CBPR-informed PA intervention before and during the pandemic have been described using four sub-themes: “Wavering between frustration and action,” “Shifting from only prioritizing family needs to taking control of self,” “Between isolation and social support,” and “Restricted access to health-related knowledge vs. utilizing internalized knowledge.” The themes intend to convey a juxtaposition between the participants' perceptions before and after the intervention, as well as during COVID-19.

The four themes commonly discuss how uncertain feelings experienced by women initially lead to frustration owing to lack of support. These feelings seemed to have resolved through engagement in the intervention, after which they could make more informed choices. However, when distancing led to isolation during the pandemic together with the lack of understanding about the novel COVID-19 infection and the recommendations, they developed conflicting feelings and a state of ambivalence yet again. There was a brief period of hesitance owing to their ambivalent state, following which women eventually identified their inner strengths with the support of the health promoters and other members in their group. This helped them reminisce the knowledge they gained from the intervention. They also gained understanding regarding the roles of the different public actors (whom they did not trust ahead of the intervention) and the recommendations to be followed through the health promoters, which further helped them recover and become resistant toward physical, social, and psychological effects of the pandemic through continuing to maintain their health and being physically active.

### Wavering between frustration and action

The theme wavering between frustration and action describes how the women in the group were frustrated and experienced mood swings in general. Their frustration was primarily owing to events from their past in their homelands together with their current life situation where they seldom had time to learn the language, be physically active, and get acquainted with the society. However, after participation in the intervention, they reported that the physical activity seemed to have reduced their frustration. They also started to believe that PA influenced their mental health. During the long-term follow-up, the participants were frustrated only when they did not have the opportunity to be physically active. When the pandemic emerged, the society, in general, was filled with fear and uncertainty, and the women said they were also initially frustrated. The women reported that they later reflected on the past experiences of the effect of physical activity and ensured that they were physically active in the best possible way even when restrictions were in place.

During the baseline focus group discussion, the women explained that they had an inherent tendency to be stressed often. Since they were unemployed and were overburdened by household chores, they mostly stayed at home and had little knowledge on their surroundings. They also perceived a lack of time to develop their local language skills through participating in courses offered in the city center. They experienced mood swings owing to their sedentary lifestyle, lack of social life, and impending thoughts about the conditions in their homeland. Some women also said that they were initially very lonely and sad, and they even refused to participate in the intervention and had to be motivated by the health promoters to take part. After participation in the intervention, they believed that they learnt how to focus on their health and change their lifestyle, rather than being stressed and constantly worried about their wellbeing. The women perceived the participation in the group intervention had helped them recover from their frustration and focus on their health and wellbeing.

“*Before I easily got annoyed and angry. I was insecure and afraid all the time. Since I started training with the group, I have become calm and happy. I think physical activity has unique effect on our mental health” (Post-intervention, focus group – 2b)*.

At the long-term follow-up, the participants mentioned that through participation in the intervention, their body and mind got used to being physically active that if they ever lacked opportunities to being active and were idle, it started to affect their mental health, and so they continued to be physically active even in the absence of group activities.

“*After participating in the group activity, I have become accustomed to being active and exercising. If I do not do it, I start to feel sad and frustrated” (Long term follow-up, focus group – 3d)*.

Women who previously suffered from mental health problems, particularly anxiety, believed that their condition worsened during the pandemic and felt frustrated since they did not receive necessary help to recover. They faced a mix of emotions, including sadness, anger, and helplessness, which led to more frustration since they realized that they were heading to nowhere with their feelings.

“*I was scared, depressed. I could not do anything. Could not stand it anymore. That is how it was. I feared everything, everything seemed stressful. I got angry for nothing; I could not even go out to get help” (During COVID-19, focus group– 4a)*.

The women in the group said that although they were anxious initially when the pandemic emerged, they recovered from fear and sadness through being more physically active. The women reported that the knowledge gained from participation in the intervention had always been with them as an inner resource, and with some motivation from the health promoter, it was activated during the pandemic. They also believed that mental health was related to PA and that poor psychological status led to a decrease in PA, and vice versa.

“*I have learnt from the group training that if one is sad, they cannot be physically active but if you are not physically active you do not feel happy either it is like a chain reaction. Yes, physical activity helps me to reduce my anxiety” (During COVID-19, focus group – 4c)*.

### Shifting from only prioritizing family needs to taking control of self

This theme describes how the women perceived that the mounting family duties were the reason for not being able to care for themselves. Even though the women decided to take time to participate in the intervention, they initially felt guilty for missing out on tending to their family during the time they were with the group. However, when the women participated in the intervention in company of others in a similar situation, they received support and helped each other. In addition, through participation, the women also realized that if they did not care for themselves and their own health deteriorated, they may not be able to care for their family. This motivated them further to be physically active. During the pandemic, the women said that they could share their knowledge with their family and help them be physically active. The women began to believe that they were important and that their lives were meaningful following participation in the CBPR intervention.

During the baseline focus group discussion, the women said that as a tradition, they usually perceived their family needs ahead of their own that sometimes they had little time to themselves. After participation in the intervention, the women said that their children observed a positive change in their mothers and were very happy for them.

“*I have come to understand that healthy women mean healthy family, because we tend to the family, we cook and care for our children.” (Post-intervention, focus group – 2d)*.

At the long-term follow-up, the women said that the intervention was successful only because it was designed in accordance with their needs. The women said that they were initially hesitant to participate, but they trusted the health promoters as they are more like them than others, given that they are from similar backgrounds and family circumstances and thus had a closer understanding of their individual needs. The women also mentioned that by including them and taking their views seriously, they started to feel that they were important. The women also mentioned that when others realized their importance, they themselves also started to believe that they were important.

“*It finally felt like our views were heard, that we were important and in fact I started to believe that I am important” (Long term follow-up, focus group – 3a)*.

The women in the group said that although initially it was very frightful when the COVID-19 pandemic emerged, they also came to realize that their fear was also affecting their general health in a negative way and that the consequences of it could be more severe than if they contracted the virus. The participants perceived that over time they understood that all that they could do was to be positive and follow the recommendations. They also tried to spread the positivity to their families and friends, which made them feel calm. The women mentioned that they also used the knowledge they gained from participation in the intervention and tried to replicate activities they did earlier such as lifting small weights, where they replaced weights with a bottle of pickled cucumber or a simple ball, and did the same activities they did with the group at home with their families.

“*I use two cans of pickled cucumber to train. I kept five kilos in one hand and another five kilos in the other and do some movements we learnt in the group. And then, I have a big ball that I also use and then I lie on the floor, and train. I also helped my family to be active” (During COVID-19, focus group – 4d)*.

### Between isolation and social support

This theme describes how women in the neighborhood were isolated and often lacked motivation to be physically active, but the participation in a group within a social context motivated them to be physically active. Health promoters had an important role in motivating some of the women to participate in the intervention at the beginning. However, following participation, the women themselves began motivating each other to be regular owing to the interaction and bonding established in the group. The women also reported during the long-term follow-up that the support and understanding they received in this group were absent in other similar group training sessions they had tried elsewhere. During the pandemic, the women after, an initial period of isolation and yearning, revived their contacts with their group *via* social media. They particularly trusted only this group and did not part take or trust in other sources on social media.

During the baseline focus group discussion, some of the women said that they decided to participate in the PA intervention to overcome their isolated and lonely lifestyle. They also believed it would give them an opportunity to make new acquaintances and also to do something useful for their body, instead of throwing their time to sitting idly and being depressed. The participants also mentioned that women in the neighborhood often lack motivation and were less informed about the importance of being physically active and therefore lead an idle life and needed someone like the health promoter to motivate them.

“*I do not have great desire for anything, I live alone and I am very depressed I need someone to push me all the time” (Baseline, focus group – 1c)*.

After participation in the intervention, the women believed that doing PA in a group helped them break their isolation, and it also improved their mental health. The women also said that when they felt less motivated on any occasion, the other group members started to message and motivate them on the WhatsApp group created by the health promoters for the group. The women felt meeting regularly made them more comfortable and secured in the group where they could freely share their views and discuss concerns without feeling threatened of their privacy. The women also mentioned that it was not just about being in a group but also the interaction between the group members facilitated by the health promoters. They said that they have participated in other group activities as the sewing circle where there was no interaction at all although they sat in a group.

“*I have no one in my life and was in a lot of grief, but when I started in the group and met others here, I started to be very happy. It now feels like I have a big family” (Post-intervention, focus group – 2a)*.

During the long-term follow-up, the women reported that when it was dark in late autumn and winter, they preferred organized group activities as the climate affected their mental health. They also mentioned that they did not feel motivated to train by themselves in the absence of group activities and it was making them more depressed. Some women also mentioned that despite creating their own groups, they had challenges to find a large enough place and facilitate the activities at stipulated times as health promoters had done. The women also said that they had tried other group training in the neighboring areas, but it was not the same since they did not receive the guidance and help from the coaches in those activities as the health promoters and fellow group members did during the PA intervention.

“*I have friends who want to train with me but we can not afford to rent a room and do activities, it is difficult to do it at home. It's more fun when we are many, and that is why we need activities that are organized by health promoters” (Long term follow-up, focus group – 3c)*.

The women perceived that when the pandemic emerged and the related recommendations were introduced, physical distancing led to social distancing, which contributed to feeling isolated, irrespective of age. The women said they felt captivated, and it also seemed like they were going back to being isolated as in the beginning before they participated in the intervention.

The women also said their mental health became worse because of social distancing, which was perceived as isolation, since initially they did not leave their home or meet anybody. If they later on had not decided to at least go for a walk, they believed that their mental wellbeing would have been seriously deteriorated.

“*It also got worse and worse since in the beginning I met no one. I've been alone since Corona started. I felt really bad. I felt like I was in prison. I needed to train at home myself. But, I had no desire to train myself. If I had not decided to go for a walk I would have become crazy.” (During COVID-19, focus group – 4d)*.

The women regarded social media as a means to breaking isolation among community members. The women were aware of the negative effects of being isolated from their earlier experiences and started to use WhatsApp and other social media more frequently during the pandemic as it helped them communicate and stay close to their family and friends from the neighborhood despite the physical distance. The women in the group even shared health tips and COVID-19-related information to each other *via* WhatsApp. Despite access to several WhatsApp groups that provided information on COVID-19, the women said they preferred the group created by the health promoters as it seemed more locally relevant. They also said it felt more comfortable in those groups since they knew other members, and they could freely contribute to the group and learn from each other as they did before.

“*I do not like social media and stuff like that. But I am in a group together with other women in the area who came with me to the training. We share knowledge with one and other in the group and we compete for being the first to share information, it makes it fun and I think we feel more stronger when we are learning together though we cannot meet in person.” (During COVID-19, focus group – 4c)*.

### Restricted access to health-related knowledge vs. utilizing internalized knowledge

This theme describes women's general sense of mistrust in the healthcare system as it is not culturally and contextually adapted. The women were also apprehensive about the short appointments with the nurses and doctors, thus the lack of opportunity to express their needs. Some experienced language barriers, and even those who could speak the local language were not satisfied with their contact with the healthcare. The women believed that even during the pandemic, they did not receive specific information or knowledge regarding health-related lifestyle. The women through participation in the intervention seemed to have gained much of the support they missed from the healthcare system. They trusted and believed in the health promoters who even explained the recommendations from the public health authorities. During the pandemic, the women although lacked support from the healthcare recalled their internal knowledge previously gained through participation in the intervention. They utilized this internal knowledge to maintain a healthy lifestyle despite staying indoors.

At the baseline discussions, the women in the group believed that there was a need for knowledge regarding how to protect and maintain health among citizens living in the neighborhood. They expressed dissatisfaction with the support they received from the healthcare system since the staff at the primary care and even specialist doctors did not give the necessary time and attention to providing tips to improve health based on their living conditions. They felt language was not a barrier; however, they could not understand how the Swedish healthcare system worked.

“*It is important for us to know what improves health and also controls blood sugar and hypertension which is a common problem here, nobody has told us things so clearly not even my doctor” (Baseline, focus group – 1a)*

During the baseline focus group discussion, the women said many people who had diabetes, hypertension, and muscle dystrophy were aware that PA had a positive impact on these conditions. They also said the problem was that they were not motivated to be physically active. After participation in the intervention, the women believed that they could change their health behaviors, which they previously could not despite the awareness. They also reported that the nurse at the diabetes healthcare was pleasantly surprised since they suddenly observed changes in blood sugar and blood pressure levels.

“*I have diabetes and I know its good to train but it was very difficult to change my eating habits and move my body, but now after participating in the activity I have changed everything, and my diabetes nurse is completely surprised as I have much lower blood sugar than before.” (Post-intervention, focus group – 2c)*.

The women who had visited a medical doctor during the pandemic for control of diabetes or other health ailments perceived that doctors never discussed COVID-19 and its impact on diabetes or high blood pressure. The participants felt that due to social distancing, there was a change in their own lifestyle, regarding which the doctor did not discuss further or give specific recommendations.

“*We hardly get appointments with the doctor these days for adults they only see children... I go to the doctor once a year for a referral to control my diabetes. They talked about coronavirus but nothing about exercising and eating well during these times.” (During COVID-19, focus group – 4b)*.

During the pandemic, the women believed that they received health-related information from many sources, but they thought that it was better to know about health and different ways to improve their health from someone in their circle who has a similar background as them and has tried it, and they did not believe in merely following advice from doctors. The women believed that it was better to hold on to one resource for information, although there were many channels of communication. In the group created by the health promoters, the women said that they not only participated in the group activities but also had the opportunity to discuss and understand recommendations from the authorities and their implications.

“*I am on Whatsapp only in certain groups created by health promoters with other women in the area. Here we have all the activities we used to have before even group training. This is how I also get all-important information that is summarized in a simple language. This way we get to understand what local authorities really recommend.” (During COVID-19, focus group – 4d)*.

## Discussion

The result of the study shows that the women were wavering between being frustrated and gaining relief from the frustration first owing to their own situation and later on because of the pandemic. Therefore, physical activity became a means of recovery from their wavering mental state. Furthermore, the women who initially valued prioritizing family needs over their own health started to take control of their health following participation in the intervention. The women who were initially isolated received support from the group through participation in the intervention and started to feel included. The participants initially complained that they did not receive health-related knowledge from healthcare both before and during the pandemic. However, after participation in the intervention where they could discuss and reflect with other group members, they believed that they gained knowledge, which also became useful, especially during the pandemic. Thus, participation in a CBPR-informed PA intervention helped the women recover from the state of ambivalence. Furthermore, they also became more decisive, making more informed decisions by taking control of their own health and wellbeing while also helping others in their family and community. The social support received from participating in the group with the other women initiated the empowerment processes that led to behavioral change and improved health among the women. Not only did the participants change their lifestyle but also spread their knowledge to their families and friends in the neighborhood, resulting in community capacity. Empowerment was initiated by engaging in reflective dialogues and activities and specifically through the support they received from other participants in the group. Empowerment is a recurrent interpersonal process fostered by setting goals, developing self-efficacy and competence, acquiring knowledge, and taking action to achieve goals (58). The health promoters also linked the group to important institutions in the society such as healthcare and social care, which was in particular highlighted during the pandemic. Given that this is a CBPR programme, there was constant dialogue and reflection within the group, where the need for adapting activities to the pandemic situation emerged. Thus, the health promoters facilitated digital activities bringing together the women and facilitating their recovery through engaging them together in the group. This helped in building community resilience among the participants, which was even transferred to their families.

Above all, the results of this study showed that participating in the CBPR intervention, the women primarily experienced improved mental and social health in addition to positively influencing their physical health. The discussion with the women indicated that the knowledge gained through participating in the group activities together with the support from the health promoters and, most importantly, the empowerment process initiated by CBPR participation seemed to have influenced their mental health.

### Improved mental health and sustainability of the CBPR intervention

Lack of physical contact, social isolation, and physical inactivity became an added burden to the existing mental health condition due to traumatic experiences and thoughts of their homelands among the women in this study. The absence of psychosocial support, particularly from the healthcare personnel owing to language and sociocultural barriers, aggravated the situation and made them feel helpless and frustrated. After participation in the intervention, the women realized that being physically active was a means to revival from psychological stress and mental health problems and also made it a routine. Several CBPR intervention studies, especially among Latina, African-American, and Asian- American communities have also shown that in an environment built on trust, participants collectively identify their resources to regain their mental strength and recover from anxiety and mental distress (59–63). Even in the case of this study, the research team was engaged in a prolonged trust-building phase (described as step 1 in the larger program) ahead of establishing a partnership with the communities, which also contributed to a long-standing involvement of the community in this study and in the larger programme.

Furthermore, our results show that after participation in the CBPR intervention, the participants in this study seemed to have experienced poor mental health only when they stopped being physically active. Numerous studies in the past have identified the effect of PA on mental health (64–66). However, in this study, such a relation also led to long-lasting commitment to being physically active since the participants experienced the effect of physical activity on their mental health, which motivated them to continue to be physically active. It can be suggested that the praxis of knowledge and learning from participation in the intervention and sharing experiences with others in the group made this CBPR intervention sustainable. These results were also in line with previous studies assessing CBPR interventions, especially those targeting behavioral change in different populations, where sustainability was related to equitable partnership established between the community members, academicians, and other stakeholders, as well as their collective actions focusing on knowledge transfer and knowledge mobilization (47, 67–69). As suggested by Wallerstein and colleagues (22, 70), the sustainability of the CBPR intervention presented in this study was owing to the fact that the PA intervention was not only built on the needs of the community but also that the citizens were involved in the development of the intervention, including defining the goals for evaluating it. Furthermore, within the larger programme in which the current study was a part, the community members defined their problems and identified themes for promoting health, and only after this phase (described within the larger program as step 2), the other stakeholders were involved together with the citizens in the planning process.

When the pandemic emerged, it had a strong effect on the psychological wellbeing of the participants, especially in the early stages since limited information was available, and the women began feeling unsure and frustrated just as they felt prior to participation. This was also in line with the previous studies (71, 72), where fear, anger, and hopelessness were identified as the most frequent traumatic emotional responses among the general public during the initial outbreak of the COVID-19 while it was still an epidemic and not declared as global pandemic.

Although the World Health Organization had raised the importance of maintaining health and engaging in regular PA during the pandemic, especially to gain relief from the related anxiety and stress (73), the general recommendation against gathering in public places, restrictions in gyms, and training centers together with the fear of even moving out of home have been barriers to PA during the pandemic (3). However, women in this study said that based on the knowledge they gained from participation in the intervention, they realized that the best means to relieve themselves from anxiety and psychological distress caused by the pandemic was by being physically active. Although they could not initially train with their groups as before, many of them started to walk regularly, which helped them alleviate their frustration and decrease their mental stress. This is also in line with the results from a cross-sectional study in Canada, which showed that preserving mental health is a motivating factor for increased PA during the COVID-19 pandemic (74).

Furthermore, the women also reapplied the knowledge they gained from participation in the intervention to be physically active from within their own homes with the limited resources available. They replicated some of the activities they performed together as a group using household tools such as a bottle of pickled cucumber to replace training equipment. They also shared their knowledge with their families and even helped them be physically active based on what they learnt from the intervention, thus strengthening family relationships and spreading a positive spirit to their family and acquaintances at the time of crisis.

### Knowledge mobilization during the CBPR interventions

Freirean ideology promotes critical consciousness and critical thinking; in this study, the citizens, when engaged in a reflection, dialogue, and action cycle, were able to link their realities and experiences in the quest of knowledge, which led them to collectively identifying solutions and taking action (75). This is also well-aligned with the results of the current study, especially during the pandemic; the citizens had access to different information from different sources, especially through social media. There was fear of receiving misinformation and thus a lack of trust in the information, particularly when information was from unknown sources. In line with the current study, a recent study has also highlighted the challenges in the use of social media as a communication channel for health-related information during the pandemic as there was an increased possibility of being misinformed (76, 77). However, in this study, the women reportedly trusted only the group created by the health promoter together with other group members with whom they could engage in a collaborative conversation. The discussions within the group together with the health promoters helped the women assess the different kinds of information and collectively assimilate knowledge from trustworthy sources. Studies on community-engaged risk communication also reported similar results including that actively engaging communities has the potential to introduce shared creation and dissemination of health information, while it also increases the possibility to involve in local communities in determining culturally appropriate mitigation policies together with concerned authorities (78, 79). However, the sustainability aspects of these initiatives were unclear since they were not built on previously existing equitable partnerships with the community built on long-standing trust (established in step 1 of the larger program) as in the case of the current study. Furthermore, the women in this study also reported that they trusted the information provided by the health promoters since they adapted and recommunicated the health information from healthcare authorities and other governmental organizations. Previous studies have also shown that lay health promoters are culturally competent in the context and often communicate informally with the community members, thus making them more comfortable (80, 81).

### Initiating empowerment during CBPR interventions

In the beginning of this study, prior to participation in the intervention, women explained their social circumstances and culture led them to prioritize their family needs over their own. They lacked time to learn the local language or get accustomed to the context in the host country. Post-colonial theories have raised concepts such as enmeshment and familism when discussing the sociocultural practices, which are common in many Arabic families (82). Arabic women are considered to be deeply bound to their families, and the family played a central role in their life. Women become enmeshed in this situation where they are continuously working to meet family needs that they lose touch with their own needs, goals, desires, and feelings. These theories also suggest that women sense guilt when they choose caring for themselves time to time, assuming that they compromised their families and are frequently lost in the process of identifying a balance (82).

However, through participating in the CBPR intervention, the women identified the strength to rebuild themselves and understood that they needed to take care of themselves so that they could care better for their family, and thus, family became a positive motivation for enabling self-change. The women also explained the feeling of being empowered in that they felt more recognized in the society than they previously did. Previous research has also shown that interventions informed by the CBPR approach has the potential to induce empowerment since the voices of communities which are otherwise not included in traditional research are heard and also recognized (83). Furthermore, the communities take part in the decision-making process, develop critical thinking, gain autonomy over their own life, and thereby the ability to change (84–86). However, in this study, empowerment has been a means to overcome enmeshment without disrupting family dynamics or cultural orientations, but rather affecting them positively, through improving women's health and thereby giving them a better chance to care for their family. The results of this study also draw on Zimmerman's definition of psychological empowerment, which is defined as individuals' perceived control over their lives and is also, in turn, related to their level of participation in community change (87). Several CBPR interventions, especially those among migrant communities, have identified empowerment as one of the key outcomes of participation in the intervention (26, 50, 63, 88). However, what is unique about the current study in contrast to other studies is that the intervention was not part of the pre-determined programme with a well-defined goal; rather, it was co-developed by the citizens of the community.

For some women, participation in the CBPR intervention was an opportunity to being physically active and also making new acquaintances to break their isolation and be included in a social context. Through social support received from fellow group members and the health promoters from their own community, the women in this study became motivated to be physically active. The findings from this study thus highlight the role of participation in the group and the social support received from the community group as a key factor for initiating the empowerment process and thereby behavioral change. The participatory dialogues and reflection within the group helped them move from a confused or ambivalent state to a more stable state, where they could take control of their life to make informed decisions. This is also in line with Freirean ideology regarding empowerment, which suggests that participation in group action and dialogues that aim at community change also enhances participants' control over their own life as well as increases the beliefs regarding their ability to change (89). Prior CBPR studies also showed that facilitating opportunities for communities to influence their development through playing meaningful roles, providing social support, building social networks, and implementing collaborative action can lead to empowerment (90–93).

According to one study among migrant women, empowerment is described as a cyclical, interpersonal process facilitated through dialogue and reflection among a group of individuals with a similar background and interest. For example, the process of empowerment was further explained in the migrant women study as starting with an initial dialogue and reflection, primarily establishing the group goals often aiming for change, further building efficacy and competence through gaining knowledge through discussion and knowledge mobilization, and finally taking action toward reaching the set goals (94). This cycle seems also well-aligned with proceeding of the events in the current study, where the women met in a group with others from the same context facing similar problems and had mutual goals to improve their health and being physically active, they gained knowledge from each other and motivated each other and finally made a change in their lifestyle by becoming physically active with the support of the group. Social support was a motivating factor to be physically active among women in this study since many women could not train by themselves in the absence of group activities during the long-term follow-up.

During the pandemic, the women perceived to be mentally stronger when being connected to the group, despite the distance, since they felt encouraged, motivated, and cheered each other, which helped them live through the acute situation. The women could communicate freely and did not feel threatened about their identity in the group, given the already established relationship with the other group members during the intervention. This strengthened the women and helped them maintain health while also promoting recovery from stress owing to the pandemic and thereby also increased community resilience. These results support the recommendations by the European Union in the OECD report, lifting the need to integrate COVID-19-related prevention work to existing local initiatives based on mutual trust to maximize the reach to communities that are frequently not covered by larger efforts at a population level (7). Prior CBPR studies have also shown that social connectedness may become foundations for recovery from natural and manmade disasters such as wars (26, 95, 96). Furthermore, earlier studies also show the role of social support or social connectedness and its relationship to community health which was facilitated by active community engagement.

The findings of this study were also in line with previous research on disaster management highlighting the role of social capital (social connectedness) in an environment built on trust, where community members bond with each other and further link with societal organizations, resulting in increased community resilience during acute situations (97). This study shows that the CBPR process increased social connectedness and led to individual empowerment, which over time may have led to community empowerment and increased community resilience during an ongoing pandemic such as COVID-19. Community resilience is regarded as the collective ability of a neighborhood to cope with stressors and efficiently return to the rhythms of daily life through a collaborative initiative built on social support following an adverse event such as a natural disaster or pandemic (95, 97).

### LHPs act as brokers building community capacity during the CBPR intervention

The local health promoters played a vital role in engaging the women in the PA intervention. In line with the guiding principles of CBPR, such as inclusion, the LHPs initially motivated and brought women together in the community who were otherwise isolated and lacked social contact to participate in the activities. It was also important that the LHPs were part of the community with whom they worked, which can be resonated in relation to the theories of situated learning by Etienne Wenger. According to Wenger, knowledge is situated and embodied in practice, and in this case, it also includes the sociocultural understanding, which results in building inclusive communities (54). The LHPs have also been instrumental in facilitating dialogue with the citizens, with an aim of creating a common understanding of problems in the neighborhood and also relating to the practices specific to the community in question. Having been trained in reflections from the works of Freire (19), and participatory methods, the LHPs individually supported the women who experienced challenges and uncertainties by introducing them to the social context, which helped them recover and identify their own strengths. Furthermore, they worked with the group as a whole to build trust and establish an equitable partnership with the research team and other stakeholders and thereby involving the women in the collective decision-making process. They also facilitated dialogues within the group while acting as bridges between the authorities who have a direct implication on the everyday lives of the community such as the public health authorities, governing bodies, and healthcare staff. All this was possible because of the LHPs' ability to empathize with their fellow community members and also because they followed and adhered to the actions they were promoting. The role of the LHPs as brokers mediating community engagement and facilitating CBPR interventions has also been discussed in previous international studies (98–101); however, they are not as common in the Swedish setting.

One of the COVID-19-related recommendations from the World Health Organization was the need to promote health behavior and PA (102). However, the women in this study said that during their diabetic control visits at the primary care, the healthcare personnel did not discuss the importance of healthy lifestyle or being physically active during the pandemic. Although several studies have highlighted that PA has decreased significantly during the pandemic (3, 103, 104), no study to date has assessed the role of healthcare staff in the context of changed lifestyle during the pandemic. Despite the lack of support from the healthcare system, the health promoters reminded the women about the knowledge they had gained through participation in the intervention. The women believed that a mere text message reminder was sufficient for them to recollect themselves and train from home.

### Community engagement through the CBPR intervention

In this study, three key aspects were basis for facilitating community engagement. First, at the start of the larger programme in which this study was a part, future workshops (described as step 2 in the larger program) were conducted ahead of the CBPR planning with other stakeholders (described as step 3 in the larger program). This gave sufficient time for the communities themselves to define the problem and even reach to an open agreement regarding strategies to improve health. Second, the community members were also part of the development of the physical activity intervention and were even actively involved in the planning of the activities together with the lay health promoters. Third, the lay health promoters in this study having a diverse role were involved in the active learning of Freire's participatory method on empowerment, which included support in facilitating the group processes contributed to an increased community engagement.

### Implication of the evaluation of the CBPR intervention

Continuous evaluation of the intervention was deemed necessary, given that the context, environmental factors, and even the people are continuously changing. Newer families have been moving into the neighborhood, and more women have expressed their interest in joining the groups, resulting in the need for more training sessions during the week. During the long-term follow-up and ahead of the pandemic, many women expressed challenges to train alone in the absence of the group, particularly in winter when they lacked motivation to go outdoors. Thus, the group activities have been readapted to suit the seasons of the year with more indoor activities during winter and additional walking and trekking activities during summer. Yet another example was the pandemic that necessitated the activities to be moved digitally. Some participants also needed digital support to learn to use applications such as Zoom and WhatsApp.

## Limitations

In addition to giving a unique insight on the impact of a CBPR intervention, the current study also takes into account the COVID-19 perspective. The main constraint in this study was that most of the discussions were held in Arabic and were translated to Swedish by the health promoters, and the audio recordings were later transcribed verbatim and again translated back to English for the purpose of analysis and presentation in the article. Such back and forth translation of data could have resulted in translation and interpretation bias, which may affect the trustworthiness of qualitative data. However, the authors of this study have cautiously handled the data. The health promoters who translated Arabic to Swedish were fluent in both the languages. In addition, all the four authors of this study were bilingual in that they could speak both Swedish and English. The analysis was performed after prolonged engagement with the data, which enabled understanding of intricate and implicit reflections of the women's experiences. Furthermore, the analyzed data were presented and discussed with the health promoters and participants to ensure no misunderstanding of the actual views.

## Conclusions

A CBPR-informed PA intervention empowered women from a disadvantaged neighborhood to become physically active and remain physically active even during a novel pandemic. Thus, the intervention seemed to have had a positive effect on how women coped with both chronic diseases and newly emerged infectious diseases such as COVID-19. Furthermore, it can be concluded that community-based resources, particularly social support and trust, are critical for promoting wellbeing and resilience among communities living in disadvantaged neighborhoods. Future research must focus on developing community-based initiatives catering to local needs by actively engaging citizens in the research process and thus empowering communities to become resilient in the face of emerging crisis.

## Data availability statement

The original contributions presented in the study are included in the article/supplementary material, further inquiries can be directed to the corresponding author/s.

## Ethics statement

The initial part of this study was approved by Regional Ethical Committee in Lund (DNR 2018/384) and the focusgroups that happened during the COVID-19 pandemic was approved by the Swedish Ethical Review Authority (DNR 2020-04063). The patients/participants provided their written informed consent to participate in this study.

## Author contributions

RR and MR moderated the focus group discussions. AK and EC had the role of observers during the focus group discussions. RR, EC, and MR analyzed the data independently and later discussed it together. AK read the transcripts and reconfirmed the findings. RR wrote the first draft of the manuscript. All authors participated in the design of the study, revised, read, and approved the final version of the manuscript.

## Funding

This study was part of a larger programme supported by Vinnova (DNR 2016–00421, 2017–01272), but the funding was primarily directed toward the establishment of a health promotion platform and did not support research presented in this study. The focus groups conducted during the COVID-19 pandemic was supported by Malmö University (FO 2020/299).

## Conflict of interest

The authors declare that the research was conducted in the absence of any commercial or financial relationships that could be construed as a potential conflict of interest.

## Publisher's note

All claims expressed in this article are solely those of the authors and do not necessarily represent those of their affiliated organizations, or those of the publisher, the editors and the reviewers. Any product that may be evaluated in this article, or claim that may be made by its manufacturer, is not guaranteed or endorsed by the publisher.
